# Special educational needs provision and academic outcomes for children with teacher reported language difficulties at school entry

**DOI:** 10.1002/jcv2.12264

**Published:** 2024-07-23

**Authors:** Sarah Griffiths, Laura Lucas, Debbie Gooch, Courtenay Frazier Norbury

**Affiliations:** ^1^ Psychology and Language Sciences University College London London UK; ^2^ School of Psychology University of Surrey Guildford UK; ^3^ Department of Special Needs Education University of Oslo Oslo Norway

**Keywords:** academic attainment, language disorder, school placement, special educational needs, statutory assessment

## Abstract

**Background:**

Language ability predicts academic attainment across the curriculum. Teacher report of language difficulties may therefore help schools identify children that require Special Educational Needs (SEN) provision. Special Educational Needs provision is intended to enable children to reach their academic potential, however the effectiveness of this for children with language difficulties is unknown.

**Methods:**

We linked teacher‐ratings on a brief language difficulties questionnaire (13‐item) collected in the first year of primary school (*N* = 7013), with data on SEN provision until age 12–13 and scores on statutory assessments at ages 5–6, 6–7 and 10–11 years from the National Pupil Database (UK). We conducted a preregistered analysis to (a) test the association between teacher‐reported language difficulties and later academic outcomes, (b) identify predictors of subsequent SEN provision for monolingual children with language difficulties and (c) test whether SEN provision is associated with better academic outcomes for these children.

**Results:**

Teacher‐reported language difficulties predicted achievement in phonics (*r*s > 0.41), reading (*r*s > 0.38), writing (*r*s > 0.32) and maths (*r*s > 0.40) assessments up to 7 years later. For those with language difficulties, having an existing diagnosis of a neurodevelopmental condition or sensory impairment was the strongest predictor of SEN registration (*OR* [95% *CI*] 8.33 [4.12, 19.24]) and special education placement (*OR* [95% *CI*] 18.89 [9.29, 42.01]) during primary school. However, 38% of children registered as having a primary speech, language and communication need, lost this registration during transition to secondary education. We could not estimate the effect of SEN provision on academic outcomes, as the majority of children with high propensity to receive SEN provision did receive provision, and very few children in SEN settings completed statutory assessments.

**Conclusions:**

Teacher perceptions of language difficulties at school entry, in the presence of additional risk factors, should prompt SEN provision. Recognition and support for language difficulties should be sustained throughout children's education.

## Graphical abstract

A pre‐registered analysis of data from a large population cohort found scores on a brief teacher‐reported language difficulties measure at school entry predicted performance on statutory tests throughout primary school. Most children with teacher‐reported language difficulties went on to have a registered Special Educational Need but provision was inconsistent and dropped off at key transition points. Short teacher‐report measures of language difficulties can help identify children requiring SEN support alongside other indicators.

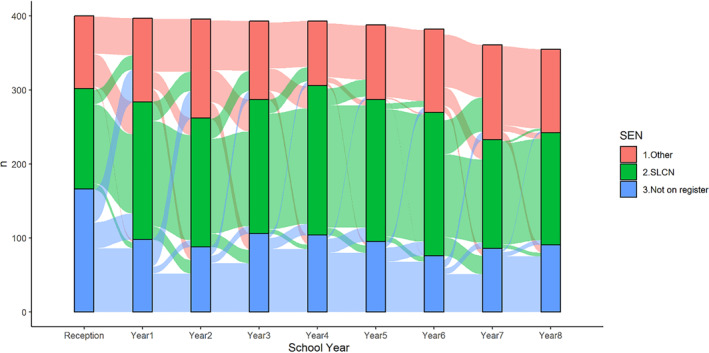

**Key points**

- Children with language difficulties are at risk of academic underachievement and may require SEN provision.
- In a pre‐registered analysis of data from a large population cohort, scores on a brief teacher‐reported language difficulties measure in the first year of school predicted performance on statutory tests throughout primary school.
- Most children with teacher‐reported language difficulties went on to have a registered SEN at some‐point in their educational journey, but provision was inconsistent and dropped off at key transition points.
- Short teacher‐report measures of language difficulties at school entry can help identify children requiring SEN support alongside other indicators.
- Recognition of, and support for, language difficulties should be sustained throughout children's educational journeys.

## INTRODUCTION

Language difficulties are a common feature of many DSM‐5 neurodevelopmental conditions, including autism, intellectual disability and language disorder (American Psychiatric Association & Association, 2013). Developmental language disorder (DLD) is the consensus term for difficulties learning, producing and comprehending spoken language that are not associated with another developmental condition (Bishop et al., 2017). Developmental language disorder is a common, but often undiagnosed, condition affecting 7.5% of children at the start of primary school (Norbury et al., 2016). Language difficulties are associated with a range of negative social, emotional, and behavioural outcomes, including academic underachievement (Yew & O’Kearney, 2013; Ziegenfusz et al., 2022).

### Language at school entry as a predictor of academic attainment

Receptive and expressive language are key for engaging in standard classroom activities and therefore for accessing the school curriculum. Direct language assessments predict achievement across the curriculum including in mathematics (Purpura et al., 2011; Vukovic & Lesaux, 2013) and literacy (Bleses et al., 2016; Pace et al., 2019). The majority of children with language disorders fail to meet academic milestones in the first year of primary school, and are more likely to leave without qualifications at the end of secondary school (Conti‐Ramsden et al., 2009; Johnson et al., 2010). Transdiagnostic language difficulties may therefore be a useful indicator of children who require additional educational support. At a group level, parent‐reported language difficulties at age 4–5 or 6–7 years have been shown to reliably predict lower scores on National Tests for reading, writing, spelling, grammar, and numeracy throughout primary years (McLeod et al., 2019). Teacher‐report of language difficulties is easier to obtain and may be more closely related to academic achievement. Our first research question in the current study was; to what extent are scores on a short teacher‐report measure of language difficulties taken in the first year of primary school (Norbury et al., 2016, 2021), associated with outcomes on statutory tests across the curriculum during primary education?

We additionally ask whether teacher‐report of language difficulties are a stronger predictor of academic outcomes for monolingual children compared to children that speak English as an Additional Language (EAL). Teacher‐reported language measures will identify language difficulties that are the result of developmental disorders but also language difficulties that are the result of lack of exposure to the community language, for example, in those who are learning English as an additional language. Around 20% of children entering school in the UK have EAL (Office for National Statistics, 2022a). These children have poorer academic outcomes in early primary education compared to peers with English as their first language (Whiteside et al., 2017), but largely catch‐up by the end of secondary school (Demie & Strand, 2006; Strand et al., 2015). Teacher report of language difficulties may therefore be a weaker predictor of future academic challenges in children with EAL. To test this, we compare the strength of the association between teacher reported language difficulties and academic outcomes for children with and without EAL.

### Determinants of special education provision for children with language difficulties

In the UK, children identified by their teachers as falling behind their peers academically are registered by their school as having a Special Educational Need (SEN) and should receive SEN support at school. Only 3.8% of pupils have Speech Language and Communication Need (SLCN) listed as their primary category of need (Office for National Statistics, 2022a). This figure is well below global prevalence estimates of DLD which range from 6.4% to 8.6% (Calder et al., 2022; Wu et al., 2023). Many children with language difficulties may therefore not receive appropriate SEN provision. More than 50% of children that meet criteria for DLD do not receive any SEN provision in the first year of school (Norbury et al., 2016). Children with more specific or profound learning and/or behavioural difficulties should receive an Education, Health and Care Plan (EHCP), which may include attending a special school or a specialist unit within a mainstream school. Only a minority of children with a registered primary SLCN (22% in 2021/22; Office for National Statistics, 2022b) receive this enhanced level of support, and only 8% of children meeting criteria for DLD receive this enhanced level of support in the first year of primary school (Norbury et al., 2016). Our second research question was, what determines the level of special education provision that children with teacher‐reported language difficulties receive during primary education?

Children that have a diagnoses of a neurodevelopmental condition associated with language disorder, such as autism, may be more likely to receive special educational services relative to children with language disorder not associated with another diagnosis (Bitterman et al., 2008; Dockrell et al., 2019). Dockrell et al. (2019) compared hours of specialist support for children with autism and DLD and found that children with autism received more hours of speech and language therapy and school support than those with DLD, even after controlling for children's behavioural and language needs. Children with language difficulties who do not have a diagnosis of another condition may therefore be less likely to receive specialist support in schools, due in part to poor identification and diagnosis of DLD (McGregor, 2020).

Regardless of diagnostic label, severity of language and communication needs, poorer academic attainment and behavioural functioning are associated with SEN provision for both children with DLD (Dockrell et al., 2019) and children with autism (McDonald et al., 2019; Wei et al., 2013; White et al., 2007). For example, Dockrell et al. (2019) found that higher ratings on the strengths and difficulties questionnaire were associated with more hours of school support for children with a diagnosis of either DLD or autism, although this was no longer the case once diagnostic status was accounted for.

Additionally, socio‐economic status (SES) and ethnicity have been shown to determine receipt of special education provision in the USA, although the exact nature of the association differs between studies. Some studies have found that children from lower SES families are more likely to receive certain types of support, such as transport to school, suggesting effective allocation of services (Wei et al., 2013). In contrast, Sturm et al. (2021) found that controlling for other factors, children from more affluent, white or middle eastern American families are more likely to receive SEN provision, suggesting that social capital and financial resource may make it easier to navigate a complex system and advocate for access to special education resources. Finally, age within a cohort has also been found to be associated with SEN provision, with the youngest children in each year being more likely to be registered as having an SEN (Anders et al., 2010).

In addition to testing what predicts special education provision for children with language difficulties, we also describe the educational journeys these children make as they move through the school system. Little is known about how receipt of SEN provision for children with language difficulties changes over time. The few studies we have suggest that there may be a lack of consistency in the allocation of placements in specialist schools of children with language disorders (Howlin et al., 2000). For example, Durkin, Simkin, Knox, et al. (2009) found that although the majority of children with language disorder (84%) consistently had a statement of SEN (equivalent to EHCP), 41% had a change in their placement type during their time at school. This study exclusively recruited children who were already in a special language unit at age 7, which may not reflect the experience of most children with language disorder who start school in mainstream provision. We therefore take an exploratory descriptive approach to look at the type and timing of SEN registration and placements in special settings for children starting school with language difficulties.

### Academic outcomes for children with language difficulties receiving special educational needs provision

The aim of SEN provision is to reduce the impact of children's learning disability on their academic outcomes, by providing an optimum environment for learning. Nonetheless, children with early identified language disorder attending special schools or receiving SEN support in mainstream schools, perform more poorly on academic assessments than children with early identified language disorder not receiving special education (Durkin, Simkin, & Knox, 2009). This likely reflects that fact that the determinants of SEN provision receipt (e.g. severity of disorder, comorbid conditions) also affect academic outcomes, and SEN provision does not fully mitigate these effects.

Several studies have attempted to deal with confounding variables which predict both receipt of special provision and academic outcomes using propensity score analysis (Austin, 2011), in which children are matched on characteristics that are predictive of SEN receipt before comparison. However, even after controlling for propensity to receive special education, academic outcomes are better for those in mainstream classrooms (Dempsey & Valentine, 2017; Kvande et al., 2019; Sullivan & Field, 2013). This may be due to accommodations in special educational settings that reduce the academic content of activities (Harrison et al., 2013), or peer effects, whereby children learn more from having peers with better language skills relative to their own (Justice et al., 2014). To address our third research question, we sought to use propensity score analysis to test whether children with teacher identified language difficulties differ in their academic outcomes based on whether they receive SEN provision.

### The current study

The current study uses data from the Surrey Communication and Language in Education Study (SCALES), combined with data from the UK National Pupil Database on academic attainment during the primary school period, along with records of special educational provision from the first year of primary school (age 4–5) up to the second year of secondary school (age 12–13). We aimed to address the following research questions:

1. Do teacher reported language difficulties at school entry predict academic performance at key assessments points throughout primary school?
2. For children with teacher‐reported language difficulties at school entry, what predicts receipt of special education provision during primary school?
3. Do children with teacher reported language difficulties at school entry that go on to receive SEN provision in primary school have different educational outcomes at the end of primary school compared to those that do not receive SEN provision?

## MATERIAL AND METHODS

### Participants

Eligible participants were all children in the first year of state‐maintained school in the county of Surrey, UK, in the 2012–2013 school year. The language difficulties questionnaire was completed for 7267 children (59% of the total population) by approximately 281 teachers in 161 state‐funded schools (156 mainstream and 5 special schools). Children were aged between 4 years 9 months (57 months) and 5 years 10 months (70 months) at the time of screening. The screen was completed for 3553 girls (49%) and 3714 boys (51%). We analysed data from all children we were able to match via anonymous pupil identification numbers to data from the National Pupil database (*N* = 7013, 97%).

### Teacher report of language difficulties

Teachers completed a short version of the Children's Communication Checklist 2 (CCC‐2; Bishop, 2003) when children were in Reception (age 4–5). The CCC–S contains the 13 items from the original CCC‐2 that best discriminated children with language disorder from their peers (Norbury et al., 2004). Seven items describe a child's communication strengths, and six describe communication deficits. Teachers rated how often the child displed each using a 4‐point scale. All 13 items were summed to create a total CCC–S score (maximum = 39), with higher scores reflecting greater language difficulties. Raw CCC‐S scores were converted into age adjusted z‐scores using the LMS method (Vamvakas et al., 2019). Children were classified as having ‘language difficulties’ if they scored above the 86% centile of CCC‐S z‐scores. This cut‐off is more restrictive than screening tools that have been employed to identify children with language needs (cf. West et al., 2021, bottom 20% and/or teacher concern) and was chosen to identify children with likely persistent language needs.

### Academic outcomes

Data from the Surrey Communication and Language in Education Study was linked to variables in the National Pupil Database via Unique Pupil Identification numbers. The National Pupil Database contains raw scores from statutory assessments taken in Year 2 (age 6–7) and Year 6 (age 10–11) in maths, reading and writing (termed Grammar, Punctuation and Spelling in Year 6), and the Phonics Screen administered in Year 1 (age 5–6). For the Year 6 tests, the database also provides standardised scores, which are designed to allow comparison between year groups. Here, a score of 100 is the expected level of achievement for the assessment; it should not be interpreted as the average level of achievement.

### Special educational needs provision

#### Special educational needs registration

The National Pupil Database indicates whether or not the child was included on the school SEN register for each year of schooling, from Reception (4–5 years) to Year 8 (12–13 years). For those on the register, a primary and sometimes secondary need is recorded using one of 12 category labels each year. The most common primary need categories are; (a) Speech language and communication need (SLCN), (b) Mild learning disability (MLD), (c) Autism Spectrum disorder (ASD), (d) Specific learning disability (SPLD) and (e) Social emotional and mental health (SEMH; previously called “Behavioural, emotional and social disability (BESD)”). The other 8 less common categories were collapsed into an “other” category in the current analysis. Details of the labels assigned to the other category can be found in Supporting Information S1.

#### Education in a special setting

The National Pupil Database provides information on whether each child was in a special school for each academic year. Additionally, it is recorded whether or not children in mainstream schools are in a special unit and whether they are in resourced provision. Children in special units receive most of their teaching in special classes within mainstream school, whereas children in resourced provision receive most of their teaching in mainstream classes but with dedicated specialist provision provided. For the purposes of the current analysis, we categorise children as either being in receipt of some form of specialist setting provision (special school, special unit or resourced provision) or being taught exclusively in mainstream classrooms.

### Other potential predictors of special educational needs provision recorded in the first year of primary education

#### Behavioural problems

Teachers completed the Strengths and Difficulties Questionnaire (SDQ; Goodman, 1997) to assess children's behaviour in the first year of school. The SDQ is a commonly used measure with good psychometric properties (Stone et al., 2010). The SDQ includes five subscales each with five items: emotional symptoms, conduct problems, hyperactivity, peer problems, and prosocial behaviour. Here we use the total score as an indicator of whether the child was deemed to have broadly defined behavioural difficulties.

#### Academic performance

Teachers completed the Early Years Foundation Stage Profile (EYFSP) as a measure of academic attainment in the first year of primary education. This is a statutory assessment that is typically completed by teachers at the end of the first year of school. The EYFSP askes teachers to rate children's progress as Emerging, Expected or Exceeding expectation in 17 areas: (a) Self‐confidence and self‐awareness, (b) Managing feelings and behaviour (c) Making relationships, (d) Moving and handling, (e) Health and Self‐care, (f) Listening and attention, (g) Understanding, (h) Speaking, (i) Reading, (j) Writing, (k) Numbers, (l) Shape, space and measures, (m) People and communities, (n) The world, (o) Technology, (p) Exploring and using media and materials and (q) Being imaginative. Children are awarded 1 point for emerging, 2 points for expected and 3 points for exceeding target expectations.

#### Diagnoses of neurodevelopmental disorders or sensory impairments

Teachers reported whether children had any existing developmental disabilities or sensory impairments. Developmental disabilities or sensory impairments included: autism, hearing/visual impairment, Down syndrome, epilepsy, neurological impairment, cerebral palsy, intellectual disability, neurofibromatosis and Noonan syndrome.

#### Demographic variables

Schools provided information on children's gender, date of birth and home post‐codes. Children born between May and August were classified as being “summer‐born”. Home post‐codes were used to look up neighbourhood deprivation rank scores (Income Deprivation Affecting Children Index; IDACI). The IDACI rank is based on the number of children in families receiving means tested benefits in each neighbourhood in England.

### Analysis plan

Our analysis plan was preregistered on the Open Science Framework in advance of accessing National Pupil Database data (osf.io/t4m8g). Our first hypothesis was that (a) teacher ratings of language difficulties would predict scores on statutory assessments in Year 1 (age 5–6), Year 3 (age 7–8) and Year 6 (age 10–11), and (b) that the strength of these relationships would be weaker for children with English as an additional language. To address this hypothesis, we estimated bivariate correlations between teacher ratings of language difficulties and scores on the Year 1 (age 5–6) and Year 2 (age 6–7) and Year 6 (age 10–11) statutory assessments. We ran analyses separately for EAL children and monolingual children, and compared the correlation strengths using Fisher's Z tests. *p*‐values were adjusted using the Bonferroni correction to account for the seven different academic outcome measures.

Our second hypothesis was that male sex, greater teacher‐rated behavioural problems, poorer language, additional diagnoses, summer birthday, poorer attainment on the EYFSP and greater socio‐economic deprivation would predict (a) SEN registration and (b) receipt of special education provision, in monolingual children with language difficulties at school entry. To address this hypothesis, we took the subset of monolingual children with teacher rated language difficulties and conducted two multiple logistic regressions. Continuous variables were standardised before modelling to aid interpretation of odds ratios.

Our final hypothesis was that there would be a difference in academic outcomes at the end of primary education for those children registered with a SEN between Year 1 and Year 6 (age 10–11) compared to those not registered, and for those who received provision in a specialist setting between Year 1 and Year 6 (age 10–11) compared to those exclusively taught in mainstream settings. We planned to use propensity score matching for this analysis. Propensity score matching requires allocation to treatment (SEN provision) to occur after measurement of potentially confounding variables (e.g. severity of language difficulties) so that the treatment cannot influence confounding. We therefore had to limit our analysis to children that did not have SEN provision in Reception year (age 4–5) when the confounding variables were measured.

Unfortunately, it was not possible to conduct the planned propensity score analysis for the following reasons; (a) for the SEN registration analysis, the majority of participants identified as having language difficulties that were not registered as having an SEN in Reception (age 4–5) were registered as having a SEN at some point between Year 1 (age 5–6) and Year 6 (age 10–11). It was therefore not possible to find suitable control participants with language difficulties who had a high propensity to receive provision who had not received any provision. We report means and confidence intervals for the statutory assessment scores at age 10–11 for the two unmatched groups, with the caveat that any differences are likely due to confounding variables. (b) For the comparison of children who did and did not receive special provision between Year 1 and Year 6 (age 10–11), there were fewer than 10 children (<30%) in the language difficulties group who started receiving special provision between Year 1 and Year 6 (age 10–11) and had also completed statutory tests in Year 6 (age 10–11). It was therefore not possible to conduct propensity score analysis with this group due to insufficient statistical power, or to report descriptive statistics due to the potential for participant identification.

## RESULTS

### Participants

Of the 7013 study children we matched to the National Pupil Database, teacher ratings of language difficulties were available for 7008 children. 1087 (15.5%) of these children had scores above the cut‐off for language difficulties. Table 1 provides descriptive statistics for each group. The language difficulties group has a higher proportion of males, children of non‐white ethnicity and children with teacher reported diagnosis of a developmental or sensory impairment in Reception (age 4–5). Children in the language difficulties group also came from more socio‐economically deprived areas and had more teacher‐rated social, emotional and behavioural challenges and poorer academic attainment in Reception (age 4–5). Children in the language difficulties group scored approximately 1 standard deviation below the mean for children with typical language across all statutory assessments, were more than 3 times as likely to have a SEN registration, and more than 15 times as likely to have attended education in a specialist setting at some point between Reception (age 4–5) and Year 6 (age 10–11).

**TABLE 1:** Shows descriptive statistics for children identified as having language difficulties in Reception (age 4–5) and those with typical language.

		Language difficulties	Typical language
	N	1050	5957
	Male *N* (%)	698 (66.48%)	2871 (48.2%)
	Ethnicity white *N* (%)	824 (78.48%)	4954 (83.16%)
	English as an additional language *N* (%)	110 (10.48%)	628 (10.54%)
4–5 years	Age (months) *M* (*sd*)	64.31 (3.55)	64.15 (3.56)
	Socio‐economic status (IDACI) *M* (*sd*)	19,576 (8014)	21,989 (7693)
	Behavioural problems (SDQ) *M* (*sd*)	14.89 (8.14)	6.57 (6.07)
	Academic attainment (EYFSP) *M* (*sd*)	26.51 (6.34)	36.91 (6.96)
	NDC diagnosis *N* (%)	211 (20.10%)	228 (3.83%)
5–6 Years	No test data *N* (%)	74 (7.05%)	147 (2.47%)
	Phonics *M* (sd)	23.44 (11.94)	33.03 (7.28)
6–7 Years	No test data *N* (%)	35 (3.33%)	238 (4%)
	Reading *M* (*sd*)	13.51 (4.46)	18.02 (3.22)
	Writing *M* (*sd*)	12.08 (4.01)	16.2 (3.21)
	Maths *M* (*sd*)	13.77 (4.11)	17.55 (3.08)
10–11 years	No test data *N* (%)	189 (18%)	605 (10%)
	Reading *M* (*sd*)	100.72 (8.48)	107.55 (6.94)
	Writing *M* (*sd*)	101.14 (8.38)	108.09 (7.38)
	Maths *M* (*sd*)	100.25 (7.84)	106.11 (6.65)
4–11 year	Special needs register in primary school *N* (%)	714 (68%)	1128 (18.94%)
	Special setting in primary school *N* (%)	82 (7.81%)	30 (0.50%)

*Note*: For categorical variables, we present raw Ns and percentages. For numeric variables, we present means and standard deviations. NDC = neurodevelopmental condition or sensory impairment reported by teacher, SDQ = strengths and difficulties questionnaire, EYFSP = Early Years foundation stage profile. IDACI = Income Deprivation Affecting Children Index Note: Year 6 (age 10–11) test score values are not standardised score and should not be interpreted as such. Department for Education set 100 as the ‘expected’ level of achievement. However, the average score is higher than 100. Note also that there is a higher proportion of children not entered for tests in Year 6 (age 10–11) in the language difficulties group. These children would likely have lower scores.

Research question 1 Does teacher rated language at school entry predict academic performance during primary school?

Table 2 provides correlation coefficients for associations between teacher rated language difficulties in the first year of school and academic outcomes for statutory assessments at age 5–6 (Phonics), age 6–7 (reading, writing and maths), and age 10–11 (reading, writing and maths), for monolingual and children with English as an additional language. Small‐moderate negative correlations were found between teacher rated language difficulties and all seven academic outcome measures. The strength of correlations did not differ between monolingual and children with English as an additional language (*p*s > 0.30).

**TABLE 2:** Correlation coefficients and 95% confidence intervals for correlations between teacher rated language difficulties at school entry and scores on statutory assessments for children with and without English as an additional language.

Age	Statutory assessment test	Monolingual	English as additional language
5–6 Years	Phonics	−0.42 [−0.44, −0.40]	−0.41 [−0.47, −0.34]
6–7 years	Reading	−0.5 [−0.52, −0.48]	−0.46 [−0.52, −0.40]
	Writing	−0.49 [−0.51, −0.47]	−0.47 [−0.53, −0.41]
	Maths	−0.46 [−0.48, −0.44]	−0.43 [−0.49, −0.37]
10–11 years	Reading	−0.38 [−0.40, −0.36]	−0.39 [−0.45, −0.32]
	Writing	−0.36 [−0.38, −0.34]	−0.32 [−0.39, −0.25]
	Maths	−0.40 [−0.43, −0.38]	−0.40 [−0.46, −0.33]

Research question 2 What predicts receipt of special education provision for monolingual children with language difficulties at school entry?

Table 3. Presents descriptive statistics for the predictor variables for those that were and were not registered with SEN, and for those who were and were not in a special setting during primary education. Table 4 provides the results of logistic regression models predicting the likelihood of being registered with a SEN and for receiving education in a special setting during primary school. The strongest predictor of both SEN registration and provision in a specialist setting was having a diagnosis of a developmental or sensory impairment in Reception (age 4–5). The next strongest predictors were academic attainment and teacher rated language skills. Teacher rated behavioural difficulties was a predictor for education in a specialist setting but not special needs registration. Whereas male gender and lower SES were predictors for SEN registration but not provision in a specialist setting. Season of birth did not predict either SEN registration or provision in a specialist setting.

**TABLE 3:** Shows the descriptive statistics for all monolingual children with language difficulties split first by whether they appear on the Special Educational Needs (SEN) register and then by whether or not they received education in a special setting at any point during primary education (age 5–11).

	Special educational needs registration		Provision in a special setting	
	No	Yes	No	Yes
*N* (%)	296 (31%)	644 (69%)	863 (92%)	77 (8%)
Male gender *M* (%)	173 (58.45%)	453 (70.34%)	563 (65.24%)	63 (81.82%)
Summer born *N* (%)	78 (26.35%)	238 (36.96%)	287 (33.26%)	29 (37.66%)
Diagnosis of NDC *N* (%)	<10 (<3%)	185 (28.73%)	126 (14.60%)	67 (87.01%)
Language difficulties (CCC‐S) *M* [95% *CI*]	1.31 [1.30, 1.32]	1.52 [1.51, 1.53]	1.42 [1.41, 1.43]	1.80 [1.76, 1.83]
Behavioural problems (SDQ) *M* [95% *CI*]	11.61 [11.23, 12.00]	16.1 [15.77, 16.43]	13.91 [13.65, 14.17]	23.38 [22.47, 24.29]
Academic attainment (EYFSP) *M* [95% *CI*]	31.08 [30.80, 31.36]	24.88 [24.64, 25.11]	27.47 [27.26, 27.68]	19.73 [19.24, 20.22]
Socio‐economic status (IDACI) *M* [95% *CI*]	20,935 [20,47, 21,395]	19,430 [19,122, 19,738]	19,819 [19,551, 20,086]	20,878 [19,952, 21,804]

*Note*: For categorical variables, descriptive are raw Ns and percentages. For numeric variables, descriptives are mean and 95% confidence intervals.

Abbreviations: CCC‐S, Children's Communication Checklist Short; EYFSP, Early Years foundation stage profile; IDACI, Income Deprivation Affecting Children Index; NDC, neurodevelopmental condition or sensory impairment reported by teacher; SDQ, strengths and difficulties questionnaire.

**TABLE 4:** Shows odds ratios, 95% Confidence Intervals and *p* values from two logistic regression models; one predicting Special Educational Needs (SEN) registration at any point in primary education (age 5–11), and one predicting receipt of education in special education setting at any point in primary education (age 5–11).

	Special educational needs registration			Provision in a special setting		
*Predictors*	*Odds Ratios*	*CI*	*p*	*Odds Ratios*	*CI*	*p*
(Intercept)	0.20	0.07–0.62	**0.006**	0.00	0.00–0.01	**<0.001**
Diagnosis of NDC	8.33	4.12–19.24	**<0.001**	18.89	9.29–42.01	**<0.001**
Language difficulties (CCC‐S)	4.19	1.97–9.12	**<0.001**	3.22	1.00–10.55	0.05
Academic attainment (EYFSP)	0.38	0.30–0.48	**<0.001**	0.45	0.26–0.75	**0.003**
Behavioural problems (SDQ)	1.09	0.89–1.34	0.39	1.75	1.25–2.46	**0.001**
Male gender	1.66	1.18–2.34	**0.004**	1.58	0.76–3.42	0.23
Summer born	1.11	0.77–1.61	0.57	1.04	0.53–2.04	0.91
Socio‐economic status (IDACI)	0.82	0.70–0.97	**0.02**	1.01	0.73–1.38	0.97
Observations	928			928		
*R* ^2^ Tjur	0.283			0.432		

Abbreviations: CCC‐S, Children's Communication Checklist Short; EYFSP, Early Years foundation stage profile; IDACI, Income Deprivation Affecting Children Index; NDC, neurodevelopmental condition or sensory impairment reported by teacher; SDQ, strengths and difficulties questionnaire.

Research question 3 Educational outcomes for children with language difficulties receiving special education provision.

Here we provide descriptive statistics comparing the educational outcomes of children with language difficulties who did not have a SEN registration in the first year of primary school, but went to get a registration between second and the final year of primary school, to those with language difficulties who never received a registration. We were not able to conduct the planned propensity score analysis, therefore the differences between these unmatched groups are likely due to confounding variables. More than half (53%) of monolingual children with language difficulties that were not registered in Reception (age 4–5) did go on to receive an SEN registration at some point in primary school. The academic outcomes in Year 6 (age 10–11) for those children who did receive a registration were poorer than the academic outcomes of those children who did not, across maths, reading, and grammar punctuation and spelling assessments (Table 5).

**TABLE 5:** Shows Year 6 (age 10–11) academic outcomes for children identified as having language difficulties in Reception (age 4–5) who did not have an Special Educational Needs (SEN) registration in Reception, grouped by whether or not they were on the SEN register at any point between Year 1 (age 5–6) and Year 6 (age 10–11).

Subject		Never on SEN register (*N* = 296)	On SEN register (*N* = 334)
Maths	*N* (%)	276 (93.24%)	274 (82.04%)
	*M* [95% *CI*]	105.92 [105.55,106.29]	97.72 [97.3,98.14]
Reading	*N* (%)	276 (93.24%)	272 (81.44%)
	*M* [95% *CI*]	104.05 [103.7104.4]	97.18 [96.76,97.6]
Writing	*N* (%)	276 (93.24%)	275 (82.34%)
	*M* [95% *CI*]	104.84 [104.46,105.22]	98.6 [98.13,99.07]

We are unable to present the academic outcomes of children who received SEN provision in a special setting between Year 1 (age 5–6) and Year 6 (age 10–11), compared to those that attended mainstream settings, because only 33 children with low language moved into a special setting after Reception (age 4–5) and fewer than 10 (<30%) of these children completed statutory assessments in Year 6 (age 10–11).

### Further descriptive analyses

#### Special education needs registrations between reception (age 4–5) and year 8 (age 12–13)

**FIGURE 1:**
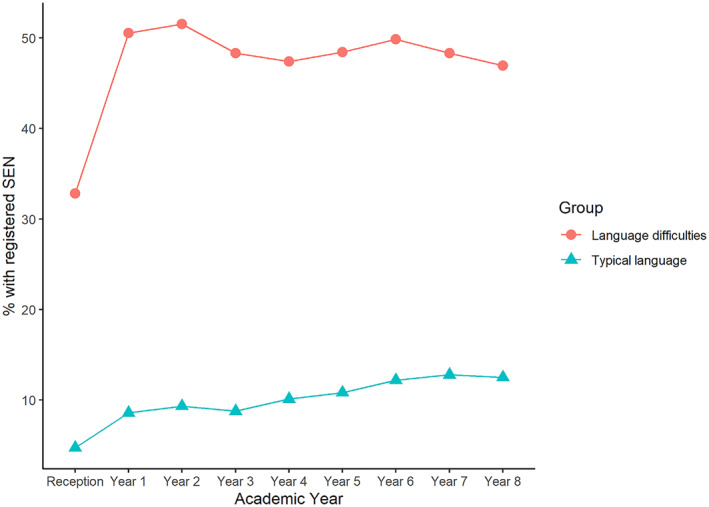
Shows percentages of children that have a registered Special Educational Needs (SEN) in each academic year, split by whether or not they were identified as having language difficulties in Reception (age 4–5).

A much larger proportion of children with teacher identified language difficulties had a registered SEN each year compared to children with typical language in Reception (age 4–5) (Figure 1). There is a large increase in SEN registrations between Reception (age 4–5) and Year 1 (age 5–6) and, in the language difficulties group particularly, there is a reduction in SEN registrations at the points where children typically transition schools (Year 2 to Year 3 and Year 6 to Year 7).

#### Type of registered need

**FIGURE 2:**
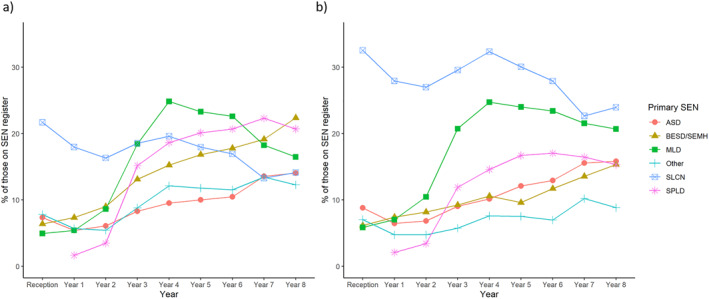
Panel (A) shows percentage of children with a registered Special Educational Needs (SEN) in each primary need category in each year. Panel (B) shows the percentage of children with teacher identified language difficulties and a registered SEN in each primary need category in each year.

We were also interested in knowing whether children with teacher reported language difficulties who were on the SEN register were registered as having SLCN or other categories of SEN as their primary need. We calculated the percentages of children with a registered SEN in each year that were in each primary SEN category. We first looked across all children in our sample and then exclusively at those with teacher identified language difficulties (Figure 2). A greater proportion of SEN children with teacher reported language difficulties in Reception (age 4–5) had SLCN listed as their primary need compared to SEN children as a whole. In both children with teacher reported language difficulties, and across the whole sample, we can see that the proportions of children with SLCN as the primary need tends to drop between Reception (age 4–5) and Year 8 (age 12–13). This is partly due to increasing absolute numbers of children in the other SEN categories but also reflects a decrease in total number of children registered as having a primary SLCN, particularly as children transition to secondary school (see Supporting Information S1 for graphs with total Ns in each category).

**FIGURE 3:**
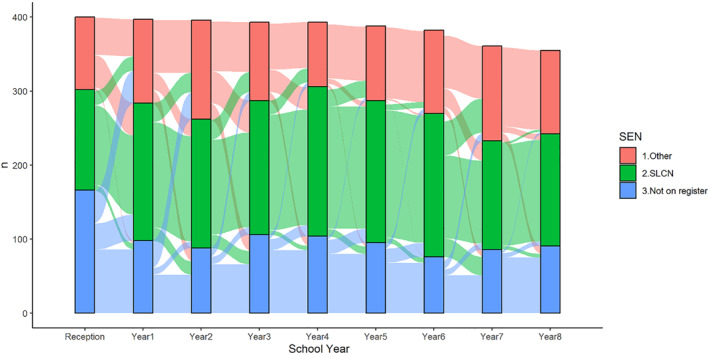
An alluvium plot showing movement in and out of the Speech Language and Communication Need (SLCN) primary need category for all children that had ever appeared in this category between Reception (age 4–5) and Year 8 (age 12–13). Each bar shows number of children with SLCN listed as their primary need, those on the register with another primary need, and those who were not on the register in that year.

In order to investigate why this drop in primary SLCN occurs, we created an alluvium plot to show the movement between primary SLCN, other primary registered need and no registered need for all children who ever appear with SLCN as their primary need between Reception (age 4–5) and Year 8 (age 12–13) (Figure 3). The greatest movement out of the SLCN category occurs at the point of transition between primary and secondary school. At this point, 38% of children with a primary SLCN in Year 6 (age 10–11) lose this categorisation in Year 7 (age 11–12). This is due to 14% losing their SEN registration completely, and the remaining 24% switch primary need category. The most common categories to switch to from SLCN are SPLD, ASD and MLD with around 6% of children with SLCN listed as their primary need in Year 6 (age 10–11) moving to each of these alternative primary need categories in Year 7 (age 11–12).

To determine whether children in the SLCN group had the most substantial language difficulties compared to those in other SEN categories, we conducted an exploratory analysis, comparing teacher reported language difficulties in Reception (age 4–5) for children in each SEN primary need category, and those with no SEN registered in Year 7 (age 11–12), across the whole cohort. Children with SLCN listed as their primary need in Year 7 (age 11–12) had the greatest teacher rated language difficulties in Reception (age 4–5) (*M* = 1.26, sd = 0.77), but children in other primary SEN groups, particularly ASD (*M* = 1.02, sd = 0.87) and MLD (*M* = 0.82, sd = 0.80), had substantially greater language difficulty scores in Reception (age 4–5) than children who were not on the register at all (*M* = −0.15, sd = 0.94) (see Supporting Information S1 for means and SD CCC‐S *z* score for each primary SEN group).

## DISCUSSION

Teacher ratings of language difficulties at school entry (age 5–6) were associated with performance on statutory educational assessments across the curriculum, including maths. This relationship was equally strong for monolingual children and children with EAL. Our findings support previous research showing the importance of oral language proficiency for effective classroom learning and academic achievement (Bleses et al., 2016; Pace et al., 2019; Purpura et al., 2011; Vukovic & Lesaux, 2013) and suggest early teacher report of language difficulties is a valid indicator of future academic attainment and SEN status.

A previous study with the same cohort found that fewer than half of the children that met diagnostic criteria for DLD (a subset of those children in the language difficulties group in this study) were registered as having SEN in the first year of school (Norbury et al., 2016). It is therefore reassuring to see that the current data show a large increase in the number of children with language difficulties who were registered as having SEN between Reception (age 4–5) and Year 1 (age 5–6), suggesting that more children with language disorder may be identified as having SEN at this point. However, rates of SEN registration tended to fall between Year 1 (age 5–6) and Year 8 (age 12–13), particularly at the points at which children typically transition between infants and junior school (Year 2 to Year 3) and between primary and secondary schools (Year 6 to Year 7).

The reduction in SEN registration during transition between schools is concerning as we know that language is a highly stable trait, and that language disorders persist into adulthood (Bornstein et al., 2018; Mawhood et al., 2000), so it is unlikely that these children's language difficulties have fully resolved. Furthermore, transition to secondary school may be a time of particular challenge for children with SEN, both in terms of navigating a more complex social environment (Hughes et al., 2013; Makin et al., 2017), and coping with an increase in the complexity of language required to access the curriculum (Snow, 2010).

The SEN primary need category listed is determined based on teacher perception of need, rather than specialist assessment of language or other skills. While a change from SLCN to another primary need does not necessarily indicate that the school no longer believe that the child has a language needs (as only one need can be listed as the primary need) it does suggest that they perceive another need to be more salient. The move away from SLCN as the primary need in secondary education may be a consequence of a reduced focus on interventions that target speech and language in secondary education (Dockrell et al., 2006). However, persistent language challenges which are not addressed can make interventions targeting other SEN such as reading or social‐emotional skills less effective as children do not have the prerequisite language skills to engage in these activities (Griffiths et al., 2021; Snowling & Hulme, 2021). Increased availability of speech and language therapy in secondary education would be beneficial in ensure that language needs continue to be recognised and supported alongside other SENs.

Reassuringly, severity of teacher‐reported language difficulties, poorer early academic outcomes and existing diagnoses known to their teachers in Reception (age 4–5) predicted subsequent SEN registration. Lower SES, which is a known risk factor for SEN (Dyson & Gallannaugh, 2008) was also a predictor of SEN registration. Children with the most severe language and social, emotional and behavioural needs in Reception (age 4–5) were also most likely to receive SEN provision in special settings, including special schools, units or resourced provision, consistent with previous reports (Dockrell et al., 2019; McDonald et al., 2019; Wei et al., 2013; White et al., 2007). After accounting for severity of language difficulties, behavioural difficulties and academic achievement, SES was not a predictor of education in a special setting, despite being a predictor of SEN registration. This pattern with SES predicting registration but not access to higher levels of support has been found previously (Dyson & Gallannaugh, 2008), and perhaps suggests that although children from lower SES backgrounds have greater needs they are not always given higher levels of support. This may be because SEN registration is primarily based on teacher judgement, whereas EHCP involves both parents and assessment by external professionals. Parents with fewer educational and financial resources may be less equipped to navigate or challenge the complex system to obtain an EHCP.

Although support is being allocated to those with the most severe needs, this appears to be inconsistent for individual children. While 70% of children with teacher‐rated language difficulties make it on to the register at some point between Reception (age 4–5) and Year 8 (age 12–13), the maximum percentage that are on the register in any given year is 50%, suggesting children frequently move in and out of SEN registration. This inconsistency is concerning as we know that language disorders are persistent and previous reports have suggested that consistency of support for children with language disorders is important for optimal outcomes (Howlin et al., 2000).

Children with language difficulties who were registered as having SEN between Year 1 (age 5–6) and Year 6 (age 10–11), had poorer academic outcomes than those who were never registered. On average, children with language difficulties group who were registered with a SEN had scores that were approximately ‐1SD below their peers with language difficulties who were not registered (see Table 5), who themselves had lower scores than children without language difficulties (see Table 1). However, as both educational attainment and severity of language difficulties in Reception (age 4–5) predict SEN registration these differences are unsurprising. Unfortunately, we were unable to carry out our intended propensity score analysis to control for these confounders, as the majority of children with teacher identified language difficulties were registered as having SEN at some point between Year 1 (age 5–6) and Year 6 (age 10–11). This meant we did not have sufficient numbers of children without SEN registration who had a high propsentity to receive registration to match with our children who were registered as having an SEN, to carry out a comparison.

### Strengths and limitations of this study

A strength of this study is that it includes a large population‐based sample of children with teacher rated language difficulties, allowing us to look at the educational outcomes and provision provided for children whose language difficulties were not necessarily formally recognised at the start of school. This is in contrast to previous studies that have looked at educational experience of just those children receiving specialist education services early (Durkin, Simkin, Knox, et al., 2009). A further strength of this study is the use of statutory assessments used by educators and policy makers, including the Phonics screen, which was introduced in the same year our cohort were eligible to take it. However, we were unable to evaluate educational success for children in special educational settings in Year 6 (age 10–11), because so few of these children took statutory assessments. While it is understandable that mainstream assessments are not accessible to all children, there is currently no standard tool for assessing academic outcomes for children unable to take these assessments, which makes it challenging to quantify the success of SEN provision.

A limitation of the current study and most previous studies assessing the outcomes of special education is that we focussed exclusively on academic outcomes. Special education has many potential benefits for children's broader development, including social and emotional development and mental health, which we were not able to evaluate. An additional limitation is that we identified language difficulties via a cut‐off on a single teacher‐report measure. This measure was designed to identify children with likely persistent language difficulties, included items that discriminate between those with and with language disorder, and is strongly associated with performance on standardised tests of language over time (Norbury et al., 2021). Given the continuous nature of language ability, language disorder can be conceptualised as representing individuals that fall at one end of a continuous distribution whose language level causes functional difficulties. Our language difficulties group therefore represent a heterogeneous group of individuals at the lower end of the distribution, some of whom have clinically significant language difficulties and some who do not. It is therefore not possible to say from this study exactly how many children with a language disorder are not receiving the educational support that they require.

## CONCLUSION

Although language screening measures in isolation rarely have sufficient levels of sensitivity and specificity to warrant universal screening (Bishop et al., 2017), our data suggest that teacher perceptions of language difficulties at school entry, in the presence of additional risk factors should prompt SEN provision. It is encouraging that most of the children identified on our teacher‐rated questionnaire did get SEN provision at some point, but this support is inconsistent and drops‐off at key transition points. Language underpins attainment across the curriculum and is an important correlate of social, emotional, and mental health. Specific support for developing language is therefore likely to be required over the longer term, particularly at transition points in the child's education journey. We recommend that educators seek to ensure that children with recognised speech and language needs in primary school continue to have these needs listed on the SEN register when they transition to secondary school alongside any additional needs that may be identified.

## DISCLAIMER (OFFICE OF NATIONAL STATISTICS)

This work contains statistical data from Office for National Statistics (ONS) which is Crown Copyright. The analysis was carried out in the Secure Research Service, part of the ONS. The use of the ONS statistical data in this work does not imply the endorsement of the ONS in relation to the interpretation or analysis of the statistical data. This work uses research datasets which may not exactly reproduce National Statistics aggregates.

## AUTHOR CONTRIBUTIONS

**Sarah Griffiths**: Conceptualization; Data curation; Formal analysis; Methodology; Project administration; Visualization; Writing – original draft; Writing – review & editing. **Laura Lucas**: Conceptualization; Writing – review & editing. **Debbie Gooch**: Data curation; Investigation; Project administration; Writing – review & editing. **Courtenay Frazier Norbury**: Conceptualization; Data curation; Formal analysis; Funding acquisition; Investigation; Methodology; Visualization; Writing – review & editing.

## CONFLICT OF INTEREST STATEMENT

The authors have declared no competing or potential conflicts of interest.

## ETHICAL CONSIDERATIONS

Consent procedures and study protocol for the screening data collection were developed in consultation with Surrey County Council and approved by the Royal Holloway Ethics Committee. UCL Research Ethics committee granted ethical approval for linking study data with data from the National Pupil Database from the (9733/005).

## Supporting information

Supporting Information S1

## Data Availability

Data from SCALES are publicly available: Norbury, C. (2022). Surrey Communication and Language in Education Study: Screening Data, 2012. [data collection]. UK Data Service. SN: 8967, DOI: 10.5255/UKDA‐SN‐8967‐1. Data obtained from the National Pupil Database are restricted and remain confidential.
