# Supplementary material for: Special educational needs provision and academic outcomes for children with teacher reported language difficulties at school entry
Source: JCPP Adv. 2024 Jul 23;5(2):e12264. doi: 10.1002/jcv2.12264 (PMC12159330; doi:10.1002/jcv2.12264)
Supplement: Supplementary file 1 — Supporting Information S1 [file JCV2-5-e12264-s001.docx]

Supplementary materials

SEN categories included in the “Other” category in the analysis include: (1) Severe learning disability (SLD), (2) Profound and multiple learning disability (PMLD), (3) Hearing impairment (HI), (4) Visual impairment (VI), (5) Multi-sensory impairment (SMI), (6) Physical disability (PD), (7) Other Difficulty/Disability (OTH) and not assessed for a specific need (NSA).


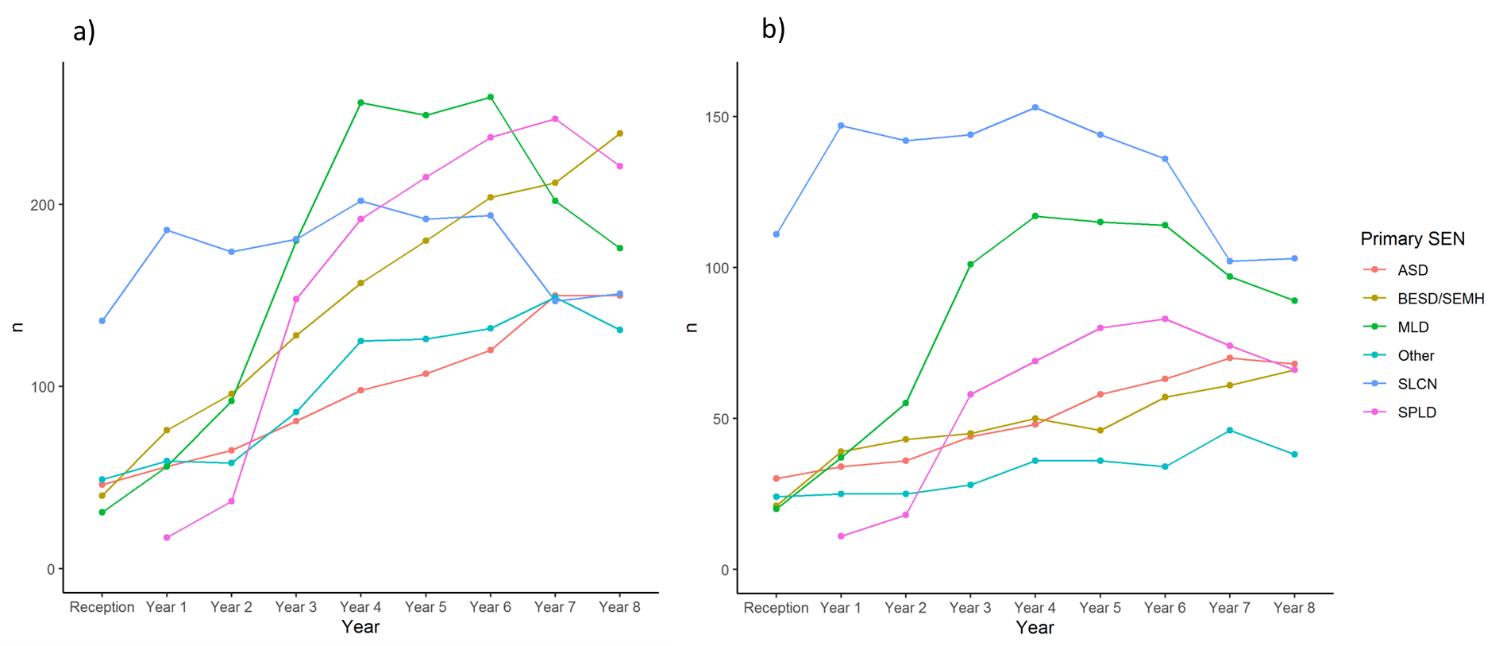


Figure S1. Panel (a) shows the number of children with a registered SEN in each primary need category in each year. Panel (b) shows number of children with teacher identified language concern and a registered SEN in each primary need category in each year.

Table S1. Mean, SD and 95% confidence intervals of CCC-S Z-scores from reception, for children in each SEN category in Year 7.

| Primary SEN category | N | Mean CCC-S | SD CCC-S | 95% CI CCC-S |
| --- | --- | --- | --- | --- |
| Not on register | 5207 | -0.15 | 0.94 | -0.16, -0.14 |
| Other | 131 | 0.52 | 0.91 | 0.44, 0.60 |
| BESD/SEMH | 204 | 0.53 | 0.89 | 0.47, 0.59 |
| SPLD | 237 | 0.58 | 0.90 | 0.52, 0.64 |
| MLD | 259 | 0.82 | 0.80 | 0.77, 0.87 |
| ASD | 117 | 1.02 | 0.87 | 0.94, 1.10 |
| SLCN | 194 | 1.26 | 0.77 | 1.20, 1.32 |
